# The practice of traditional and complementary medicine and factors associated with it among the medical staff in Malaysia

**DOI:** 10.1186/1471-2458-14-S1-O22

**Published:** 2014-01-29

**Authors:** Maihebureti Abuduli, Azam Rahimi, Nimetcan Mehmet Orhun, Zaleha Md Isa, Syed Mohamed Aljunid

**Affiliations:** 1 UNU-IIGH, UNU-IIGH Building, UKM Medical Center, Jalan Yaacob Latiff, 56000 Cheras, Kuala Lumpur, Malaysia; 2 Xinjiang Uyghur Medical College, Xinjiang, China; 3 UKM Medical Center, Jalan Yaacob Latiff, 56000 Cheras, Kuala Lumpur, Malaysia; 4 United Nations University International Institute for Global Health (UNU-IIGH), 56000 Cheras, Kuala Lumpur, Malaysia

## Background

Traditional and Complementary Medicine (T&CM) is garnering increasing interest and acceptance among the general population. Different types of T&CM treatments were increasingly applied and practised by the public but ignorance about T&CM poses a communication gap between public health and the healthcare profession. The aim of this study was to determine the practice of T&CM and factors associated with it among medical staff in five selected hospitals in Malaysia.

## Materials and methods

This study employed a cross-sectional design, which was carried out using quantitative and qualitative methods.

## Results

A total of 46.3% of the medical staff had ever used T&CM in their life and 32.5% of them used T&CM in the last one year, while 48.6% of the medical staff had ever referred T&CM to their patients or families in their life, and 25.2% of them referred T&CM in the last one year. Knowledge regarding T&CM was poor but positive perception regarding education in T&CM was high. Knowledge regarding T&CM was significantly higher in Hospital Duchess of Kent (52%, p = 0.001), among non-Malays (44%, p = 0.047), and pharmacists (47.2%, p = 0.03). Positive perception regarding education in T&CM among medical staff was higher among females (88.1%, p = 0.002) and pharmacists (93.7%, p < 0.001). There was no significant association between practice of T&CM and perception of education in T&CM.

## Conclusions

Many medical staff had not been exposed to T&CM education, however most of them had positive perception about health education/training in T&CM. The provision of information on T&CM practice and its associated factors among medical staff may help to integrate T&CM into the mainstream medicine.

